# Identification and antimicrobial susceptibility of bacteria isolated from corneal ulcers and healthy eyes of canines in Ibagué-Tolima, Colombia

**DOI:** 10.3389/fvets.2026.1755813

**Published:** 2026-05-01

**Authors:** Dunia Yisela Trujillo Piso, Yeli Camila Van-Arcken Aguilar, Mónica Yamile Padilla Barreto, María del Pilar Sánchez Bonilla

**Affiliations:** Faculty of Veterinary Medicine and Animal Sciences, Impronta Group of Research, Cooperative University of Colombia, Ibagué, Tolima, Colombia

**Keywords:** bacteria, ciprofloxacin, corneal ulcers, dogs, *Streptococcus*

## Abstract

**Objective:**

To identify the ocular microbiota of healthy dogs and with corneal ulcers, and the sensitivity-resistance of the isolated bacteria to antibiotics.

**Animal studied:**

60 dogs were included. 30 of them free of clinical or ophthalmic disease; 30 diagnosed with a corneal ulcer.

**Procedures:**

Samples for microbiological analysis were obtained by a sterile swab passed over the conjunctival sac and the cornea. The samples were inoculated on agars and identified by microscopy, Gram stain, and biochemical tests. Sensidiscs of sulfa, ciprofloxacin, tobramycin, tetracycline and gentamicin were included for the antibiogram.

**Results:**

67% of the patients with corneal ulcers showed bacterial growth; 83% were Gram-positive bacteria. *Staphylococcus* spp., *Streptococcus* spp., and *Pseudomonas* spp. were the most frequently isolated bacteria and were mainly sensitive to ciprofloxacin, followed by tobramycin, sulfa drugs, gentamicin, and tetracycline. 33% of the healthy patients showed bacterial growth with predominance of Gram-positive bacteria. The bacterial genera mainly isolated were *Staphylococcus* spp., *Bacillus* spp. and *Acinetobacter* spp., which in turn were sensitive to ciprofloxacin, sulfa drugs and gentamicin, and resistant to tetracycline and tobramycin.

**Conclusion:**

*Staphylococcus* was the most frequently isolated genus in healthy and dogs with corneal ulcers. In corneal ulcers, *Streptococcus* and *Pseudomonas* also predominate. The bacteria isolated in dogs with and without corneal ulcers were mainly sensitive to ciprofloxacin, hence its use is recommended in these patients, and the use of tetracycline and gentamicin is advised against due to their significant resistance results.

## Introduction

The ocular microbiota of dogs consists of a wide variety of microorganisms that inhabit the corneal-conjunctival surface and the tear film. Under specific circumstances, such as corneal ulcers, these microorganisms (primarily bacteria) may opportunistically invade the cornea and trigger infection (1).

Aerobic Gram-positive bacteria are the most commonly found in the healthy canine eye and include *Staphylococcus spp., Streptococcus spp*., and *Bacillus spp*. Some Gram-negative bacteria may also be present in the conjunctival sac of dogs, with *Pseudomonas spp*. being the main microorganism isolated within this group (2). Metagenomic studies suggest the presence of rare Gram-negative bacteria in healthy eyes, including species such as *Ralstonia spp., Burkholderia spp., Cupriavidus spp*., and *Sphingomonas spp*. (3).

Identifying bacteria in canine corneal ulcers can help reduce sequelae and complications associated with their presence, as several studies indicate that these conditions involve changes in the conjunctival and corneal microbiota. Such alterations may progress to collagenolytic ulcers or melting corneal disease, requiring special consideration regarding antibiotic therapy (4).

Studies worldwide highlight the need to characterize microbiota in specific regions or countries, as evidence suggests that microbial composition may vary by geographic location and under particular conditions or disorders. This situation also underscores the importance of determining antibiotic susceptibility and resistance patterns of bacteria involved in these processes to prevent complications (1, 5–7).

Ekapopphan et al. in Taiwan reported *Staphylococcus spp*., followed by *Pseudomonas aeruginosa*, as the most frequently isolated bacteria in corneal ulcers, with the former showing resistance to fluoroquinolones (5). Studies in the USA reported isolation of *Staphylococcus pseudintermedius* (22%), *Staphylococcus epidermidis* (12%), *Staphylococcus capitis* (11%), and *Pseudomonas aeruginosa* (10%). The combination of neomycin, polymyxin, and bacitracin, together with ofloxacin or amikacin, proved most effective in controlling bacterial growth (8). Isolated case reports from China and the United States describe rare bacterial species in canine corneal ulcers, such as *Moraxella canis*, resistant to ciprofloxacin (9), and *Salmonella infantis*, sensitive to tobramycin and ofloxacin (10).

The present study aims to identify the ocular microbiota of healthy dogs and those with corneal ulcers, as well as the antibiotic susceptibility and resistance patterns of the isolated bacteria used in Ibagué, Tolima, Colombia.

## Materials and methods

This study included 60 dogs of various breeds, ages, and both sexes. Thirty of them had no clinical or ophthalmic disease, while the remaining 30 were diagnosed with unilateral corneal ulcer. The study was approved by the Bioethics Committee of National Bioethics of the University of La Salle (Consecutive Protocol 166), and samples were collected with informed consent from the owners of the dogs.

All dogs underwent a complete clinical and specialized ophthalmic examination, which included slit-lamp biomicroscopy, Schirmer tear test, fluorescein staining, and tonometry. The healthy group consisted of dogs without evidence or recent history of systemic disease. Dogs with a positive fluorescein test were included in the corneal ulcer group and received therapeutic management after sample collection for microbiological analysis.

Samples for microbiological analysis were obtained by passing a sterile Amies swab (Amies Agar Gel with Charcoal Transport Swabs – BBL™), previously moistened with sterile saline, through the lower conjunctival sac toward the third eyelid, avoiding contact with eyelid margins for the healthy group. For dogs with corneal ulcers, the swab was passed over the cornea and conjunctiva with the same precautions. Samples were immediately transported to the veterinary microbiology laboratory at Cooperative University of Colombia.

### Microbiological culture and antibiotic susceptibility testing

For isolation and identification of microorganisms, samples were inoculated using the surface streaking technique with a calibrated loop on Blood Agar and MacConkey Agar plates (TM-MEDIA, Rajasthan, India). Plates were incubated for 24–48 h at 37 °C under aerobic conditions. Bacterial genus and species identification was performed through culture examination using microscopy and classical methods, including Gram staining, morphology, and biochemical tests (BBL Crystal identification systems, BD, Sparks, MD, USA).

Antibiotic susceptibility of the isolated microorganisms was assessed using the Kirby-Bauer disk diffusion method on Mueller-Hinton agar (TM-MEDIA, Rajasthan, India), following the guidelines established by the clinical and laboratory standards institute (CLSI Pennsylvania, USA). A portion of the identified bacterial culture was inoculated into 5 mL of Brain Heart Infusion (BHI) (TM-MEDIA, Rajasthan, India) broth in a tube adjusted to a turbidity equivalent to the 0.5 McFarland standard (10^8^ CFU/ml). A sterile cotton swab was dipped into the inoculum, excess liquid was removed by pressing the swab against the inner wall of the tube above the broth level, and the sample was uniformly streaked across the entire surface of a Mueller-Hinton agar plate (TM-MEDIA, Rajasthan, India) in four directions (mass inoculation). Plates were allowed to dry for approximately 5 min before antibiotic disks were placed on the agar surface using sterile forceps and gently pressed. Plates were left at room temperature for 15 min to allow complete diffusion of antibiotics, then incubated at 37 °C for 18–24 h in a bacteriological incubator. Inhibition zones were measured with a ruler under reflected light against a dark background.

The antibiotic disks used were: tobramycin (10 μg), ciprofloxacin (5 μg), trimethoprim-sulfamethoxazole (25 μg), gentamicin (10 μg), and tetracycline (30 μg) (Beckton Dickinson Diagnostics BD SENSIDISC™, Sparks, MD, USA). Cultures were classified as susceptible (S), intermediate (I), or resistant (R) based on inhibition zone diameters. Interpretative criteria were as follows:

- Trimethoprim-sulfamethoxazole: resistant ≤ 10 mm, Intermediate 11–15 mm, Susceptible ≥16 mm.
- Tobramycin: resistant ≤ 12 mm, Intermediate 13–14 mm, Susceptible ≥15 mm.
- Ciprofloxacin: resistant < 15 mm, Intermediate 16–20 mm, Susceptible ≥21 mm.
- Tetracycline: resistant < 14 mm, Intermediate 15–18 mm, Susceptible ≥19 mm.
- Gentamicin: resistant < 12 mm, Intermediate 13–14 mm, Susceptible ≥15 mm.

## Results

A total of 60 dogs were included in the study: thirty presented with corneal ulcers and were treated at the veterinary clinic of the Cooperative University of Colombia in Ibagué, and thirty healthy dogs served as the control group. Dogs of any age, breed, sex, and reproductive status were eligible. In both groups, 50% were male and 50% female.

**Figure 1:**
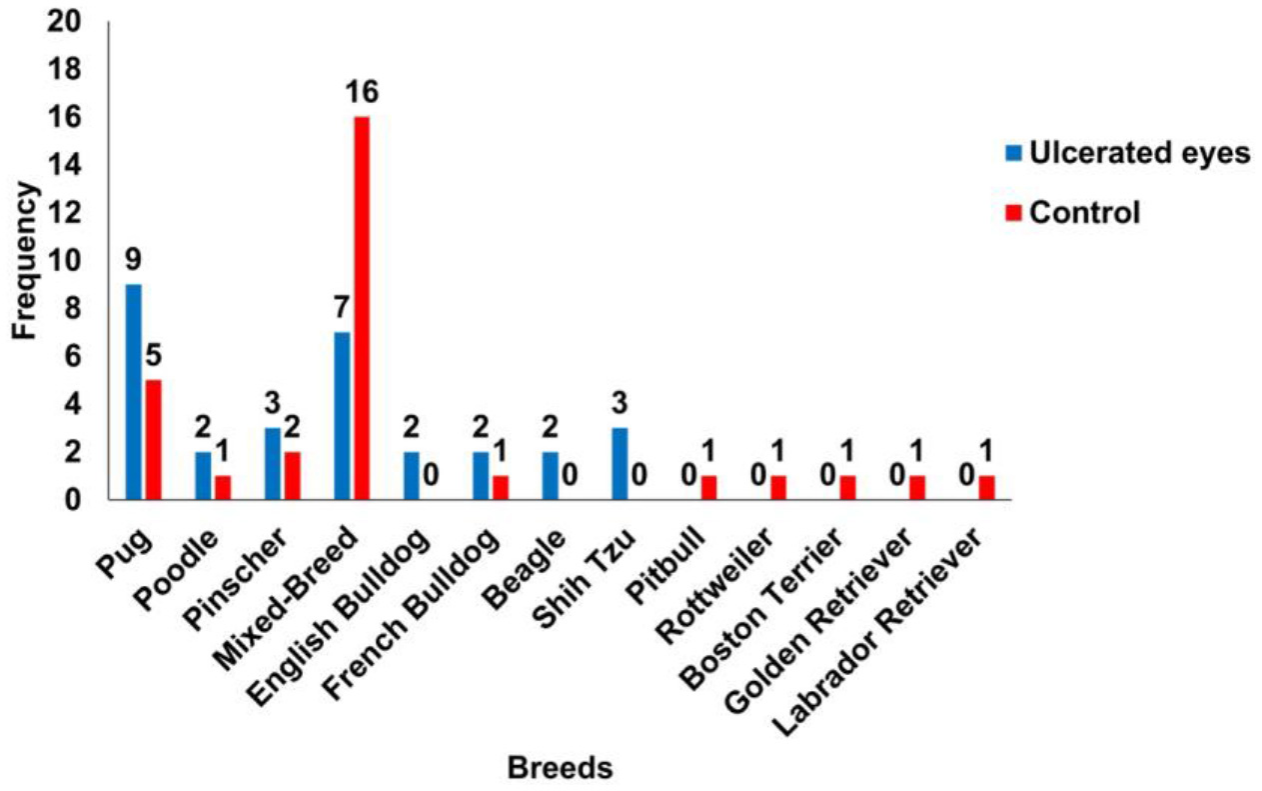
Breeds of dogs included in the study. Note the high frequency of mixed-breed and Pug dogs in both groups.

In the healthy group, 53.3% were mixed-breed (16/30), 16.7% were Pugs (5/30), and Pinschers accounted for 6.7% (2/30). Breeds such as Pitbull, Poodle, Rottweiler, Boston Terrier, French Bulldog, Golden Retriever, and Labrador each represented 3.3% (1/30). Among dogs with ulcers, 30% were Pugs (9/30), followed by mixed-breed dogs at 23.3% (7/30), Pinscher and Shih Tzu at 10% each (3/30), and Beagle, Poodle, French Bulldog, and English Bulldog at 6.7% each (2/30) (Figure 1).

The mean age of dogs in the control group was 4.12 years (*SD* = 3 years), with a minimum age of 2 months and a maximum of 14 years. In the ulcer group, the mean age was 4.67 years (*SD* = 3.83 years), ranging from 4 months to 16 years.

In the ulcer group, 33.3% (10/30) showed no bacterial growth, while microorganisms were detected in the remaining 67%. A single microorganism was isolated in 56.6% of cases (17/30); two microorganisms were identified in 6.6% (2/30), and four microorganisms were isolated in one patient (3.3%).

**Figure 2:**
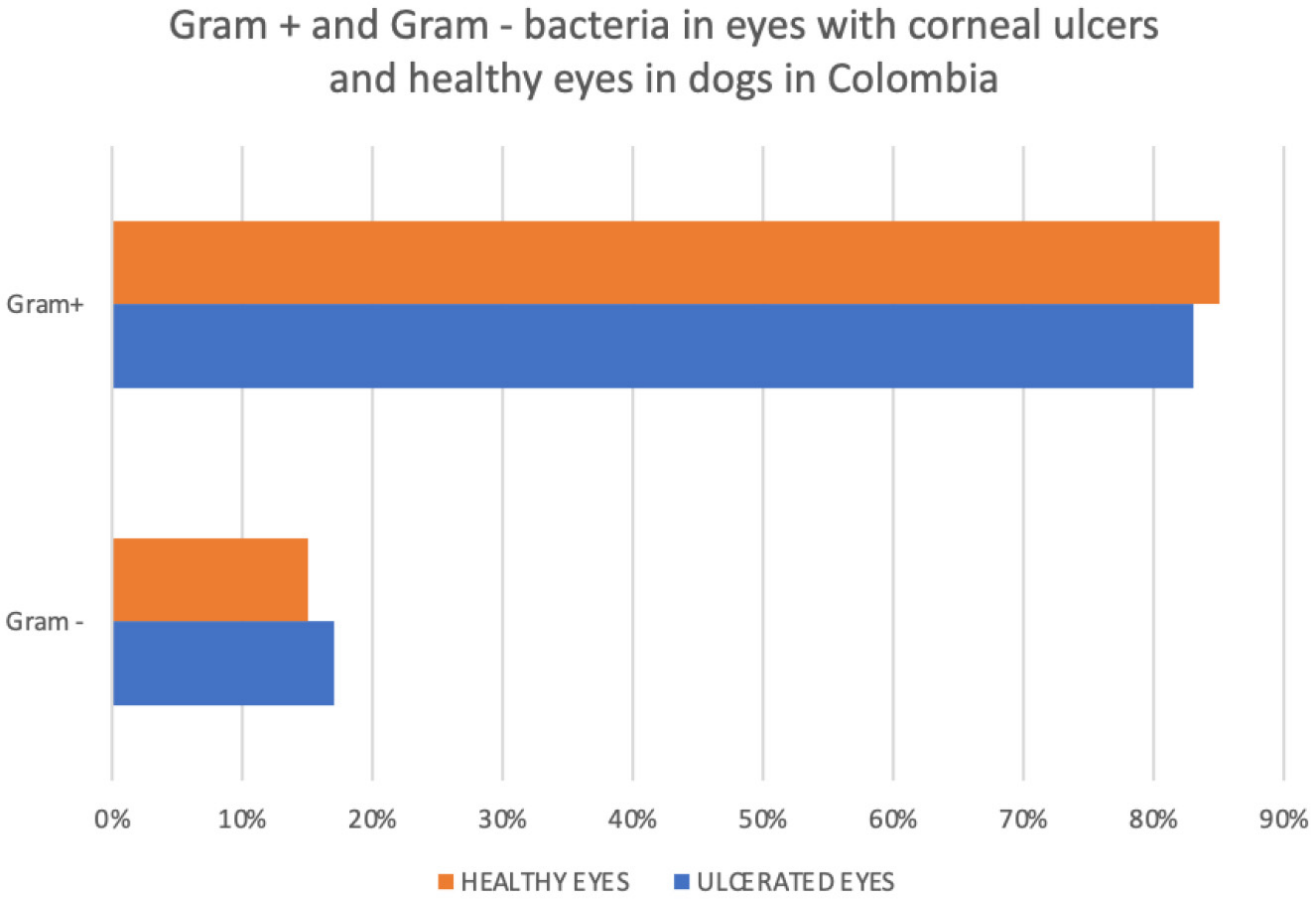
Gram-positive and gram-negative bacteria associated with canine ocular microbiota and corneal ulcers.

A total of 24 bacterial colonies were isolated, of which 83% (20) were Gram-positive and 17% (4) were Gram-negative (Figure 2).

**Figure 3:**
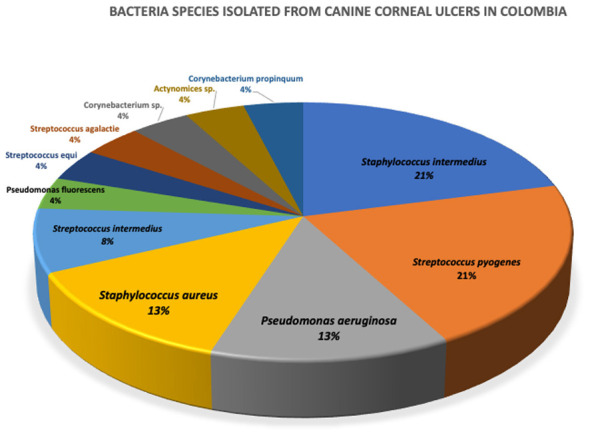
Bacteria species isolated from canine corneal ulcers in Ibagué, Tolima, Colombia.

The predominant Gram-positive species were *Staphylococcus intermedius* (21%) and *Streptococcus pyogenes* (21%), followed by *Pseudomonas aeruginosa* (12.5%), *Staphylococcus aureus* (12.5%), *Streptococcus intermedius* (8.3%), and others such as *Streptococcus equi spp. equi, Pseudomonas fluorescens, Actinomyces spp., Corynebacterium propinquum, Streptococcus agalactiae* (Group B), and *Corynebacterium spp*. (4.2%) (Figure 3).

**Figure 4:**
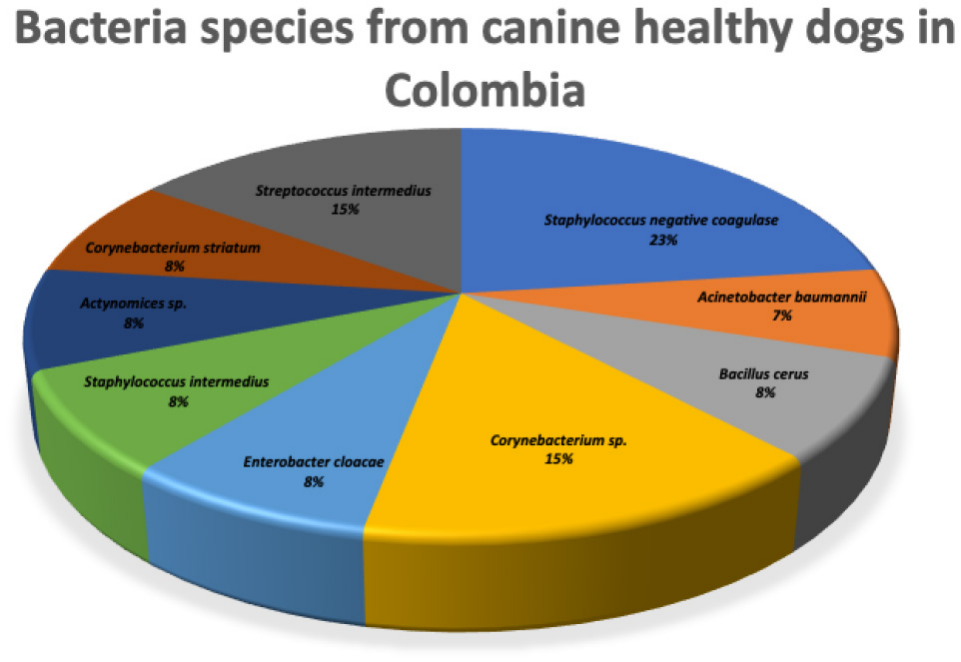
Bacteria isolated from healthy canine eyes in Ibagué, Tolima, Colombia. Coagulase-negative Staphylococcus was the predominant species.

In healthy dogs, 53.3% (16/30) showed no microbial growth. A single bacterium was identified in 43.3% (13/30), and two bacteria were isolated in one dog (3.3%). Most isolates were Gram-positive (85%), with only two Gram-negative isolates (15%). Nine bacterial species were identified. The predominant species was coagulase-negative *Staphylococcus* (23%), followed by *Streptococcus intermedius* and *Corynebacterium spp*. (15.4%). Other species included *Actinomyces spp., Corynebacterium striatum, Bacillus cereus, Staphylococcus intermedius, Enterobacter cloacae* (Group B), and *Acinetobacter baumannii*, each with a frequency of 7.7% (Figure 4).

Antibiotic susceptibility testing was performed on all isolated microorganisms using sulfamethoxazole-trimethoprim, tobramycin, ciprofloxacin, tetracycline, and gentamicin. In the control group, isolates such as *Actinomyces spp., Corynebacterium striatum*, and *Streptococcus intermedius* could not be tested due to insufficient sample size. Similarly, in the ulcer group, *Corynebacterium spp*. and *Streptococcus agalactiae* were excluded for the same reason, and *Candida albicans* was not tested as it is not a bacterium. Antibiogram results are shown in Table 1.

**Table 1:** Antimicrobial resistance profile of isolated microorganisms in healthy eyes and ulcerated eyes in dogs from Ibagué, Tolima- Colombia.

Group	Bacteria	Sulfa trimethoprim (R ≤ 10 mm, Int 11–15 mm, S > 16mm)		Tobramycin (R ≤ 12 mm, Int 13–14 mm, S ≥15mm)		Ciprofloxacin (R ≤ 15 mm, Int 16–20 mm, S ≥21 mm)		Tetracycline (R ≤ 14 mm, Int 15–18 mm, S ≥19 mm)		Gentamicin (R ≤ 12 mm, Int 13–14 mm, S ≥15 mm)	
		(mm)	R–S	(mm)	R–S	(mm)	R–S	(mm)	R–S	(mm)	R–S
**Control**	*Acinetobacter baumannii*	8	R	13	R	24	S	9	R	21	S
	*Acinetobacter baumannii*	20	S	25	S	34	S	31	S	28	S
	*Bacillus cerus*	22	S	8	R	9	R	20	S	14	R
	*Bacillus cerus*	26	S	7	R	11	R	20	S	17	S
	*Corynebacterium* spp	25	S	24	S	38	S	22	S	25	S
	*Enterobacter cloacae*	29	S	29	S	30	S	21	S	32	S
	*Staphylococcus intermedius*	6	R	22	S	28	S	9	R	23	S
	*Staphylococcus* negative coagulase	6	R	10	R	38	S	10	R	11	R
	*Staphylococcus* negative coagulase	40	S	30	S	40	S	40	S	40	S
	*Staphylococcus* negative coagulase	31	S	19	S	24	S	31	S	28	S
	*Staphylococcus* negative coagulase	19	S	30	S	26	S	11	R	30	S
**Ulcerated eyes**	*Actinomyces* spp	15	I	7	R	9	R	15	I	11	R
	*Corynebacterium propinquum*	5	R	5	R	8	R	13	R	6	R
	*Pseudomona aeruginosa*	7	R	20	S	30	S	8	R	19	S
	*Pseudomona aeruginosa*	15	I	9	R	15	R	10	R	11	R
	*Pseudomona aeruginosa*	10	R	16	S	30	S	10	R	17	S
	*Pseudomona fluorescens*	27	S	26	S	35	S	20	S	28	S
	*Staphylococcus aureus*	16	S	8	R	16	I	5	R	8	R
	*Staphylococcus aureus*	25	S	9	R	20	I	25	S	15	S
	*Staphylococcus epidermidis*	30	S	30	S	30	S	40	S	40	S
	*Staphylococcus intemedius*	27	S	18	S	26	S	24	S	21	S
	*Staphylococcus intermedius*	19	S	20	S	29	S	6	R	6	R
	*Staphylococcus intermedius*	6	R	24	S	36	S	9	R	25	S
	*Staphylococcus intermedius*	18	S	14	I	33	S	27	S	15	S
	*Staphylococcus intermedius*	22	S	18	S	25	S	8	R	10	R
	*Streptococcus equi*	27	S	26	S	35	S	14	R	28	S
	*Streptococcus intermedius*	11	I	6	R	5	R	9	R	12	R
	*Streptococcus intermedius*	11	I	6	R	5	R	5	R	12	R
	*Streptococcus pyogenes*	11	I	11	R	10	R	9	R	13	I
	*Streptococcus pyogenes*	10	R	8	R	16	I	5	R	8	R
	*Streptococcus pyogenes*	7	R	27	S	7	R	7	R	7	R
	*Streptococcus pyogenes*	30	S	8	R	16	I	14	R	8	R
	*Streptococcus* spp	7	R	7	R	7	R	7	R	7	R

### Antibiotic susceptibility analysis

A descriptive analysis of inhibition zone diameters for each antibiotic was performed in both groups for all microorganisms.

In the control group, bacteria were predominantly sensitive to trimethoprim-sulfamethoxazole (72%), with resistance observed in only three species (28%). Sixty-four percent were sensitive to tobramycin, while 36% were resistant. Eighty-one percent of bacteria were sensitive to ciprofloxacin and gentamicin, with 19% resistant to these two antibiotics. Sensitivity to tetracycline was 63%, while 17% were resistant (Table 2).

**Table 2:** Percentages of antibiotic sensitivity and resistance in bacteria isolated from healthy canine eyes.

Antibiotic	Trimethoprim-sulfa	Tobramycin	Ciprofloxacin	Gentamicin	Tetracycline
Sensitivity	72%	64%	81%	81%	63%
Resistance	28%	36%	19%	19%	17%

Among bacteria isolated from ulcerated eyes, 45% were sensitive to trimethoprim-sulfamethoxazole, 22% showed intermediate sensitivity, and 31% were resistant. Sensitivity to tobramycin was 45%, intermediate sensitivity 4.5%, and resistance 50%. For ciprofloxacin, 45% were sensitive, 18% intermediate, and 38% resistant. Sensitivity to tetracycline was 23%, intermediate 4.5%, and resistance 72%. gentamicin showed 41% sensitivity, 4.5% intermediate, and 54% resistance (Table 3).

**Table 3:** Percentages of antibiotic sensitivity and resistance in bacteria isolated from canine corneal ulcers.

Antibiotic	Trimethoprim-sulfa	Tobramycin	Ciprofloxacin	Gentamicin	Tetracycline
Sensitivity	45%	45%	45%	41%	23%
Intermediate	22%	4.5%	18%	5%	4.5%
Resistance	31%	50%	38%	54%	72%

Descriptive statistics of inhibition zone diameters are presented in Table 4.

**Table 4:** Descriptive statistics of inhibition zone diameters for both study groups.

Group	Antibiotic	N	Mean	SD	Minimum	Maximum
**Control**	Sulfa trimethoprim	11	21.09	10.91	6	40
	Ciprofloxacin	11	27.46	10.33	9	40
	Gentamicin	11	24.46	8.45	11	40
	Tetracycline	11	20.36	10.34	9	40
	Tobramycin	11	19.73	8.88	7	30
**Ulcerated eyes**	Sulfa trimethoprim	22	16.18	8.44	5	30
	Ciprofloxacin	22	20.14	11.00	5	36
	Gentamicin	22	14.86	8.82	6	40
	Tetracycline	22	13.18	8.93	5	40
	Tobramycin	22	14.68	8.12	5	30

An independent-samples Student's *t*-test (Welch's correction) was performed to compare the mean antimicrobial susceptibility values between the Control group and the ulcerated eyes group for the different antimicrobials evaluated (sulfamethoxazole–trimethoprim, ciprofloxacin, gentamicin, tetracycline, and tobramycin). The results indicated that no statistically significant differences were observed between groups for any of the antimicrobials analyzed (*p* > 0.05). In the Control group, mean susceptibility values ranged from 19.73 ± 8.88 to 27.45 ± 10.33, while comparable mean values were observed in the ulcerated eyes group, with no evidence of relevant variations attributable to the presence of ocular ulceration. These findings suggest that, under the conditions of this study, ocular clinical status was not associated with significant changes in the antimicrobial susceptibility of the evaluated microflora.

## Discussion

Sixty percent of households in Colombia own a dog (11). Among these, mixed-breed dogs are the most prevalent in the country, which explains their inclusion in this study as the group most predisposed to corneal ulcers—surpassing brachycephalic breeds such as Pugs. Due to their cranial conformation and reduced corneal nerve density, Pugs are reported to be 19 times more likely to develop ulcerative keratitis compared to other brachycephalic breeds and have been considered the most predisposed to this condition (12, 13).

Regarding age, the findings of this study align with previous reports indicating that young dogs are more predisposed to corneal ulcers, likely due to their hyperactive nature, which increases the risk of traumatic corneal ulcers (13).

Corneal ulcers, regardless of the primary cause, are frequently contaminated by bacteria. Controlling these microorganisms is essential to prevent complications such as collagenolytic ulcers resulting from accelerated corneal tissue loss. Previous studies suggest that 70%−90% of corneal ulcers are contaminated by bacteria (2, 5, 14). This incidence was similar to our findings, where 67% of corneal ulcers exhibited bacterial growth. These results contrast only with those reported by Hewitt et al. and Verdenius et al., who observed bacterial growth rates of 46% and 59%, respectively, and attributed the lower percentages to prior antibiotic therapy before clinical evaluation (1, 15).

In both healthy eyes and those with corneal ulcers, Gram-positive bacteria predominate, although Gram-negative bacteria are also present in healthy dogs at lower frequencies (16). Morales et al. reported that Gram-negative bacteria were most frequently isolated from corneal ulcers (48.43%), with *Pseudomonas aeruginosa* being the most common species. Gram-positive genera such as *Streptococcus* and *Staphylococcus* were the second most frequently isolated (39.06%) (14). More recent studies suggest that Gram-positive bacteria are now the most commonly isolated in canine corneal ulcers, possibly indicating a shift in pathogenicity (2). These findings are consistent with our results, where Gram-positive bacteria accounted for 83 and 85% of isolates in ulcerated and healthy eyes, respectively, while Gram-negative bacteria represented 17 and 15%.

Some authors suggest that corneal ulcers may progress to collagenolytic or melting ulcers due to high growth of Gram-negative bacteria, particularly *Pseudomonas spp*., which can express proteases (15, 17). This observation aligns with our findings, as *Pseudomonas spp*. was not detected in healthy eyes (18).

In this study, 56.6% of ulcerated eyes exhibited growth of a single microorganism, similar to healthy eyes. Only a small percentage of both groups showed growth of multiple microorganisms, suggesting that corneal ulcers do not worsen due to polymicrobial infections but rather due to the pathogenicity of specific bacterial species. Ulcerated eyes yielded 24 bacterial colonies, compared to only nine in healthy eyes, indicating that corneal ulcers significantly alter ocular microbiota.

Based on our results, *Staphylococcus* and *Streptococcus* species were dominant in corneal ulcers, whereas the latter were absent in healthy eyes. *Pseudomonas spp*. was isolated exclusively from ulcerated eyes. These findings agree with those reported by Tsvetanova et al. (2021), who highlighted the high pathogenicity of *Streptococcus* species, which is associated with virulence factors conferring greater antibiotic resistance (18).

In Peru, the most recent study identified *Staphylococcus spp*. as the most frequently isolated bacterium in these patients, followed by *Streptococcus* (19). In Brazil, in 2006, the most relevant bacteria were *Staphylococcus* and *Corynebacterium* (20). In 2009, the same country reported a predominance of *Pseudomonas spp*., followed by *Staphylococcus* and *Streptococcus* in that order (14). However, the most recent Brazilian study found that *Staphylococcus* predominates in canine corneal ulcers, followed by *Pseudomonas aeruginosa*, with no report of *Streptococcus* among the isolates (2).

In Thailand (2018), the most frequently isolated bacteria were *Staphylococcus spp*. and *Pseudomonas aeruginosa* (5), while in India, the most important bacteria in corneal ulcers are *Staphylococcus*, followed by *Streptococcus*, although the latter has a very low incidence (4.17 vs. 58.33% for *Staphylococcus*) (21). Previous studies in India had reported Staphylococcus as the sole agent isolated in these cases (22, 23).

In the United Kingdom, *Streptococcus*, followed by *Pseudomonas* and *Staphylococcus*, are the most prevalent bacteria in canine corneal ulcers (24). In the United States, the most frequently isolated genera in 2020 were *Staphylococcus* (32.3%), *Streptococcus* (19.1%), and *Pseudomonas* (12.5%) (1) (Table 5).

**Table 5:** Isolated bacterias in corneal ulcers of dogs in different countries of the world. Frequency of bacterias were written in order of presentation.

**Country**	**Isolated bacteria**	**Researcher-date**
Perú	*Staphylococcus* sp. *Streptococcus* spp.	Torres-Calderón et al., (19)
Brazil	*Staphylococcus sp.* *Corynebacterium sp*.	Prado et al., (20)
Brazil	*Pseudomonas sp.* *Staphylococcus sp. Streptococcus sp*.	Morales et al., (14)
Brazil	*Pseudomonas aeruginosa*	Casemiro et al., (2)
Thailand	*Staphylococcus sp.* *Pseudomona aeruginosa*	Ekapopphan et al., (15)
India	*Staphylococcus sp.* *Streptococcus sp*.	Mahajan et al., (21)
United Kingdom	*Streptococcus sp.* *Pseudomonas sp.* *Staphylococcus sp*.	Goss et al., (24)
United States	*Staphylococcus sp.* *Streptococcus sp.* *Pseudomonas sp*.	Hewitt et al., (1)

Our findings reveal that the most important genus in canine corneal ulcers in Colombia is *Staphylococcus*, with *Streptococcus* showing an equal frequency of isolation, followed by *Pseudomonas spp*. These results highlight the growing importance of *Streptococcus spp*. in canine corneal ulcers—a genus recently reported as the second most relevant in melting ulcers, traditionally surpassed by *Pseudomonas*, and associated with severe corneal deterioration, as previously observed in horses with corneal ulcers (18, 25).

The role of *Streptococcus spp*. in ulcerative keratitis has attracted increasing interest. Although these bacteria are frequently isolated from ocular samples of healthy and diseased dogs and other tissues, it has been suggested that they possess a significant ability to metabolize fructose (26), which is abundantly available in the corneal stroma—a tissue extensively exposed in corneal ulcers. This could explain their replication in corneal ulcers, although studies remain inconclusive regarding their exponential growth in canine eyes.

Bacteria isolated from the control group in this study were highly sensitive to sulfonamides, tobramycin, ciprofloxacin, tetracycline, and gentamicin. However, our results suggest that sensitivity patterns change in the presence of corneal ulcers, with bacteria showing high sensitivity to ciprofloxacin and sulfonamides but high resistance to gentamicin, tetracycline, and tobramycin.

To our knowledge, this is the first study on ocular microbiota in dogs with corneal ulcers and healthy eyes in Colombia. We compared our results with those from other countries where similar research has been conducted, and microbiota profiles were comparable. These studies recommend antibiotic therapy with neopolybac (neomycin, polymyxin, and bacitracin) (8), ciprofloxacin, gatifloxacin, moxifloxacin, tobramycin, and gentamicin (14). Quinolones are suggested as the first-choice antibiotics for corneal ulcers. Among quinolones, ciprofloxacin was included in this study and showed the highest sensitivity rates among isolated bacteria, supporting its use and that of other fluoroquinolones such as ofloxacin as priority treatments, as suggested by Verdenius et al. (15). These antibiotics offer superior bacterial sensitivity, effective corneal penetration, and excellent tissue accumulation (27).

Ciprofloxacin, tobramycin, and gentamicin for topical use are the most accessible antibiotics in our country. Our results suggest avoiding aminoglycosides for corneal ulcers, as bacteria involved in these cases exhibit high resistance rates, as previously reported by Tsvetanova et al. (18). We also recommend performing antibiograms or combining two antibiotics in patients with suspected *Pseudomonas spp*. infections or confirmed *Streptococcus spp*. isolates, particularly in melting ulcers, since these bacteria have shown variable sensitivity to gentamicin and tobramycin and resistance in the latter species (2).

Candida albicans was found in a sample of the corneal ulcers analyzed and its finding, although not statistically significant for our study, demonstrates the importance of this pathogen in these cases, since it can act as a cause or aggravator of ulcerative keratitis and lead to corneal melting, as reported by Cho et al. (28).

## Conclusion

The bacterial genera most frequently found in healthy eyes and those with corneal ulcers in Ibagué, Tolima-Colombia were *Staphylococcus spp., Streptococcus spp*., and *Pseudomonas spp*. The high isolation rate of *Streptococcus spp*. in corneal ulcers suggests the potential role for Streptococcus in the development of corneal ulcers and this increasing prevalence could be due to environmental factors influencing the infection. Furthermore, this study indicates that bacteria isolated from corneal ulcers are primarily sensitive to ciprofloxacin, followed by sulfonamides. Therefore, in canine patients where antibiogram testing has not been performed, ciprofloxacin should be considered the initial and primary treatment option. Among the antibiotics evaluated in this study, ciprofloxacin is the most readily available in the country, making it the preferred choice to prevent severe ocular complications such as melting corneal ulcers.

## Data Availability

The original contributions presented in the study are included in the article/supplementary material, further inquiries can be directed to the corresponding author.
